# IFNΛ3/4 locus polymorphisms and IFNΛ3 circulating levels are associated with COPD severity and outcomes

**DOI:** 10.1186/s12890-018-0616-6

**Published:** 2018-03-21

**Authors:** Adrian Egli, Jyotshna Mandal, Desiree M. Schumann, Michael Roth, Brad Thomas, D. Lorne Tyrrell, Francesco Blasi, Kostantinos Kostikas, Wim Boersma, Branislava Milenkovic, Alicia Lacoma, Katharina Rentsch, Gernot G. U. Rohde, Renaud Louis, Joachim G. Aerts, Tobias Welte, Antoni Torres, Michael Tamm, Daiana Stolz

**Affiliations:** 1 Applied Microbiology Research, Department of Medicine, University of Basel, Basel, Switzerland; 2 Clinical Microbiology, University Hospital Basel, Basel, Switzerland; 3 Clinic of Pneumology and Pulmonary Cell Research, University Hospital Basel, Petersgraben 4, 4031 Basel, Switzerland; 4 Li Ka Shing Institute for Virology, University of Alberta, Edmonton, Canada; 5 Department of Pathophysiology and Transplantation, Università degli Studi di Milano, Milan, Italy; 6 Department of Pneumology, Medisch Centrum Alkmaar, Alkmaar, The Netherlands; 7 Department of Pneumology, Institute for Pulmonary Diseases, Belgrade, Serbia; 8 Department of Microbiology, Hospital Universitari Germans Trias i Pujol, Badalona, Spain; 9 Laboratory Medicine, University Hospital Basel, Basel, Switzerland; 10 Department of Respiratory Medicine, Maastricht University Medical Center, Maastricht, The Netherlands; 11 Department of Pneumology, CHU Liege, University of Liege, GIGAI Research Group, Liege, Belgium; 12 Department of Pneumology, Amphia Hospital/Erasmus MC, Breda, The Netherlands; 13 Department of Pneumology, Medizinische Hochschule Hannover, Hannover, Germany; 14 Department of Pneumology, Hospital Clinic, Barcelona, Spain

**Keywords:** Interleukin 28B, Cohort, Mortality, Biomarker, Single nucleotide polymorphisms

## Abstract

**Background:**

Interferon lambdas (IFNLs) have important anti-viral/bacterial and immunomodulatory functions in the respiratory tract. How do IFNLs impact COPD and its exacerbations?

**Methods:**

Five hundred twenty eight patients were recruited in a prospective observational multicentre cohort (PROMISE) study. The genetic polymorphisms (rs8099917 and rs12979860) within the IFNL3/4 gene region and circulating levels of IFNL3 in COPD patients were determined and associated with disease activity and outcome during a median follow-up of 24 months.

**Results:**

The GG genotype significantly influenced severe exacerbation rate (42 vs. 23%; *p* = 0.032) and time to severe exacerbation (HR = 2.260; *p* = 0.012). Compared to the TT or TG genotypes, the GG genotype was associated with severe dyspnoea (modified medical research council score ≥ median 3; 22 vs 42%, *p =* 0.030). The CC genotype of the rs12979860 SNP was associated with a poorer prognosis (body mass index, airflow obstruction, dyspnea and exercise capacity index ≥ median 4; 46 vs. 36% TC vs. 20.5% TT; *p* = 0.031). Patients with stable COPD and at exacerbation had significantly lower circulating IFNL3 compared to healthy controls (*p* < 0.001 and *p <* 0.001, respectively). Circulating IFNL3 correlated to post-bronchodilator FEV_1_%predicted and the tissue maturation biomarker Pro-collagen 3.

**Conclusion:**

IFNL3/4 polymorphisms and circulating IFNL3 may be associated with disease activity and outcomes in COPD.

**Trial registration:**

Clinical Trial registration http://www.isrctn.com/ identifier ISRCTN99586989 on 16 April 2008.

## Background

Interferons (IFN) are known to have important direct anti-viral and anti-bacterial effects, as well as potent modulatory effects on the adaptive immune response via the induction of hundreds of IFN-stimulated genes (ISGs) [1, 2] The newest discovered class of IFN, the IFN lambda (IFNL) family, has four members: IFNL1–4 [3]. The IFNL receptor consists of a heterodimer with an alpha subunit (IL28RA) and a beta subunit (IL10RB). IL10RB is ubiquitously expressed, whereas IL28RA expression is restricted and interestingly, it is highly expressed on lung epithelial cells [4] and alveolar macrophages [5]. When a virus is seen by the pattern recognition receptors which are found on macrophages and epithelial cells, IFNL gene expression is stimulated via various signalling pathways [6]. This leads to increased circulating IFNL3 which interacts with the IFNL receptor expressed on lung, intestinal and liver cells, and via the JAK-STAT signalling cascade induces interferon stimulated genes which in turn influence viral replication [6]. A series of single nucleotide polymorphisms (SNPs) in the IFNL3/4 gene region have been described [7–9] and associated with variable IFNL3/4 gene expression [10–13]. The variability of IFNL3 during viral or bacterial infections may lead to significant differences in the subsequent immune response and thus variable clinical outcomes [3].

IFNL3 has immune-modulatory and anti-tumorigenic effects and is induced by viral infections [6, 14]. Viral infections play an important role in the exacerbation of asthma [15–17] and COPD [18–20]. Reduced interferon activity during a respiratory syncytial virus infection has been linked to the later development of asthma in children [20, 21]. The mechanism of virus-induced exacerbations of COPD is not well-defined. Recently, the role of IFNLs in the exacerbation of asthma has been explored [22–24]. However, no data are available regarding the effect of IFNL3 or its polymorphisms on the exacerbation and further clinical outcomes of COPD. COPD patients exposed to rhinovirus consistently showed a trend towards less IFNL expression in bronchoalveolar lavage fluid [25] and in animal models it has been shown that IFNL plays a role in viral (influenza A, coronavirus and rotavirus) modulation [6].

We hypothesize, that in patients with COPD, SNPs in the IFNL3/4 gene will impact clinical outcomes such as exacerbation and that they might be associated with circulating markers of inflammation and tissue remodelling. Therefore, we aimed to explore the IFNL3/4 polymorphisms (rs8099917 and rs12979860) and circulating IFNL3 in association with the occurrence of exacerbation of COPD and all-cause mortality in a multinational, multicenter, prospective, longitudinal, observational cohort study of patients with clinically stable and exacerbated COPD.

## Methods

### Study overview

**Fig. 1:**
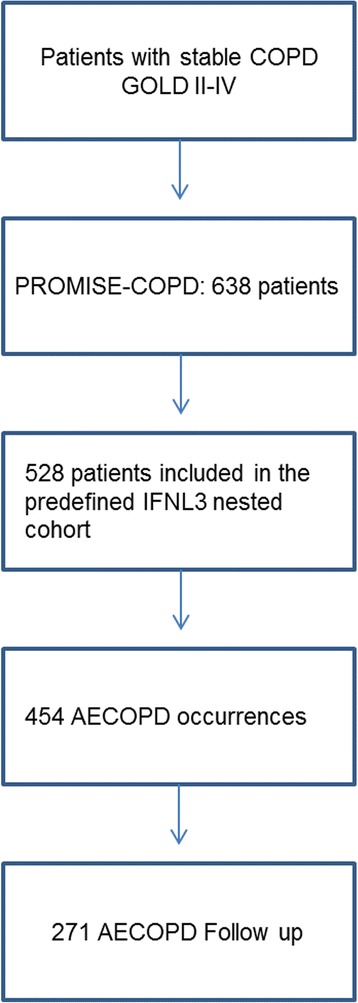
Flow chart of the patient cohort selection criteria

Patients in stable state COPD with GOLD II to IV were enrolled for an observational prospective trial (PROMISE-COPD; www.controlled-trials.com identifier ISRCTN99586989). The study details have been published previously [26]. For the current nested biomarker study, 638 patients were consecutively recruited and followed at 11 European hospital pneumology departments from November 2008 to October 2011 (Fig. 1). Details of inclusion and exclusion criteria were previously published [27]. We analysed data from 528 patients, who completed the first 6 month-follow-up of the study and for whom serum samples from visit 1 were available. We used serum samples at the stable phase and during the first episode of exacerbation of COPD.

For each patient, a physical examination was performed, vital signs were registered, and a detailed history obtained. Spirometry and 6-min walk tests (6MWTs) were performed following American Thoracic Society guidelines [24, 25]. One- and two-year follow-up in a stable phase, including a series of outcome markers were determined as previously published [26]. Additionally, patients treated for infection-triggered exacerbation of COPD had a scheduled follow-up four weeks post-exacerbation onset. Acute exacerbations were defined as an acute sustained worsening of dyspnoea, cough and/or sputum beyond normal day-to-day variations in a patient with underlying COPD; severe exacerbations were defined as an exacerbation requiring hospitalisation [28]. A total of 30 age and gender unmatched healthy controls were included in the study.

### Ethics

The study was an observational study without specific intervention. The IFNL3 genotypes and serum levels were retrospectively determined and had no impact on the treatment decisions. The study was approved by the respective participating local IRBs in all centers (Ethikkommission beider Basel EKBB/295707, Medical Ethical Committee Amphia Ziekenhuis 958, Medical Ethical Committee North Holland M08–016, Klinicki Centar Srbije Eticki Odbor, Clinical Research Ethics Committee Germans Trias I Pujol Hospital, Medizinische Hochschule Hannover Ethikkommission 5071, Ethics committee of the Hospital Clinic of Barcelona, Ethics committee of the Policlinico of Milan, Ethics committee in Greece). All patients provided a written informed consent.

### IFNL3 ELISA assay

IFNL3 serum levels were determined in the stable phase and during exacerbation of COPD. A commercially available sandwich immunoassay ELISA kit that showed no cross-reactivity with IFNL2 (IL28A) or IFNL4 (IL29; Human IL-28B/IFN-lambda 3 DuoSet ELISA, DY5259, R&D Systems Minneapolis, MN, USA) was used, according to the manufacturer’s instructions. The IFNL3 assay had a linear range of detection from 31.20–2000 pg/ml.

### IFNL genotyping

Two common SNPs, rs8099917 and rs12979860, in the IFNL3/L4 gene regions were determined as previously described [11]. The distribution of minor and major allele genotypes is comparable to previous publications in European populations [29, 30].

### Collagen markers

As previously described [31], serum levels of fragments of collagen type III (C3M), fragments of collagen type VI (C6M), pro-form of collagen type III (Pro-C3) and pro-form of collagen type VI (Pro-C6) were measured with Nordic Bioscience assays according to the manufacturer’s instructions.

### Inflammatory markers

Procalcitonin, Copeptin, pro-adrenomedullin (ADM), and atrial natriuretic polypeptide (ANP) were measured as previously described [32].

### Statistics

Differences in dichotomous variables were evaluated using the Chi-square test or Fischer’s exact test, as appropriate. Normally distributed parameters were analyzed using the Student’s t-test for equality of means. All other continuously non-normally distributed parameters were evaluated using the non-parametric Mann-Whitney U test or Kruskal-Wallis test, as appropriate. If the IFNL3 was below the detection level, the sample was assigned the value 31.20 pg/ml which is the lowest detectable value with the assay used in this study. Kaplan Meier curves were created to determine survival within 2 years and overall survival, occurrence of exacerbation and occurrence of severe exacerbation. The log-rank test was used to compare differences between survival curves. The Statistical Package for Social Sciences Program (SSPS Inc., version 22 for Windows) was used. All tests are two-tailed; a *p*-value of < 0.05 was considered significant. Results are expressed as mean (standard deviation) or median (interquartile range), unless otherwise stated.

## Results

Five hundred twenty eight patients were included in this nested study (Fig. 1). The majority of the patients were male (71%) and the average age was 66.9 years (Table 1). 70% of the patients were past smokers and 50% of the patients were classified as GOLD II.

**Table 1:** Baseline characteristics of patients included in the study

	Mean (SD), *n* (%)
Gender: Male	377 (71)
Age, years	66.9 (9.3)
Current smoker, n (%)	157 (30)
BMI (kg/m2)	26.00 (5.13)
Unadjusted Charlson Score (*n* = 528)	1.82 (1–16)
BODE index (median; IQR)	3 (1–4)
6MWT (m)	380.61 (104.69)
Exacerbation rate	2.05 (0–15)
Severe exacerbation rate	0.39 (0–8)
Lung function (post-brd)	
FEV_1_, in L	1.40 (0.71)
FVC, in L	2.81 (0.89)
FEV_1_/FVC%	47.87 (13.97)
FEV_1_, % predicted	49.89 (16.8)
FVC, % predicted	80.70 (21.21)
Collagen markers [ng/ml]	
C3M	30.54 (12.61)
C6M	15.25 (8.62)
Pro-C3	13.29 (10.03)
Pro-C6	8.76 (4.31)
EL-NE	7.78 (6.78)
GOLD Grade^a^	
GOLD II	262 (50)
GOLD III	180 (35)
GOLD IV	80 (15)
rs8099917 genotypes	
TT	339 (65)
GG	26 (5)
TG	155 (30)
rs12979860 genotypes	
CC	76 (30)
TT	45 (18)
TC	131 (52)
MMRC score (median; IQR)	2 (1–2)
Inflammation markers at baseline	
Copeptin, pMol/L	12.57 (16.66)
Adrenomedullin, nMol/L	0.69 (0.38)
Atrial Natriuretic Peptide, pMol/L	113.67 (101.03)
Procalcitonin, μg/l	0.09 (0.14)
SF-36	
Physical function	51.54 (25.94)
Role physical	51.05 (43.48)
Role emotional	66.26 (43.52)
Social Functioning	69.49 (28.56)
Mental Health	64.89 (20.07)
Body Pain	74.19 (27.94)
Vitality	51.79 (21.00)
General Health	47.88 (22.90)
SGRQ	
Symptoms score	49.30 (22.65)
Activity score	57.22 (22.90)
Impacts score	32.11 (18.66)
Total score	42.39 (18.11)

Continuous data are shown as mean (SD) or median (interquartile range) and categorical variables as No. (%). BMI = body mass index; brd = bronchodilator; BODE = BMI, airflow obstruction, dyspnea and exercise capacity; 6MWD = 6-min walk distance; C3M = fragments of collagen type III; C6M = fragments of collagen type VI; Pro-C3 = pro-forms of collagen type III; Pro-C6 = pro-forms of collagen type VI; EL-NE = neutrophil elastase-generated fragments of elastin; GOLD = Gold Initiative for Chronic Obstructive Lung Disease; MMRC = modified Medical Research Council; SF-36 = 36-item Short-Form Health Survey; SGRQ = St. George’s Respiratory Questionnaire

^a^GOLD grades are based on FEV1% predicted: 50% ≤ II ≤ 80%; 30% ≤ III ≤ 50%; and IV ≤ 30%

### IFNL3 genotyping

The distribution of both rs8099917 and rs12979860 SNPs adhered to the Hardy Weinberg Equilibrium with the χ2 test for deviation equalling 2.21 for rs8099917 and 0.781 for rs12979860. Both values were less than 3.84 which represents the 5% significance level for 1 degree of freedom and therefore the null hypothesis that the population is in the Hardy-Weinberg frequencies is not rejected [33]. The most common genotype rs8099917 TT (65%) was followed by TG (30%) and GG (5%; Table 1).

**Fig. 2:**
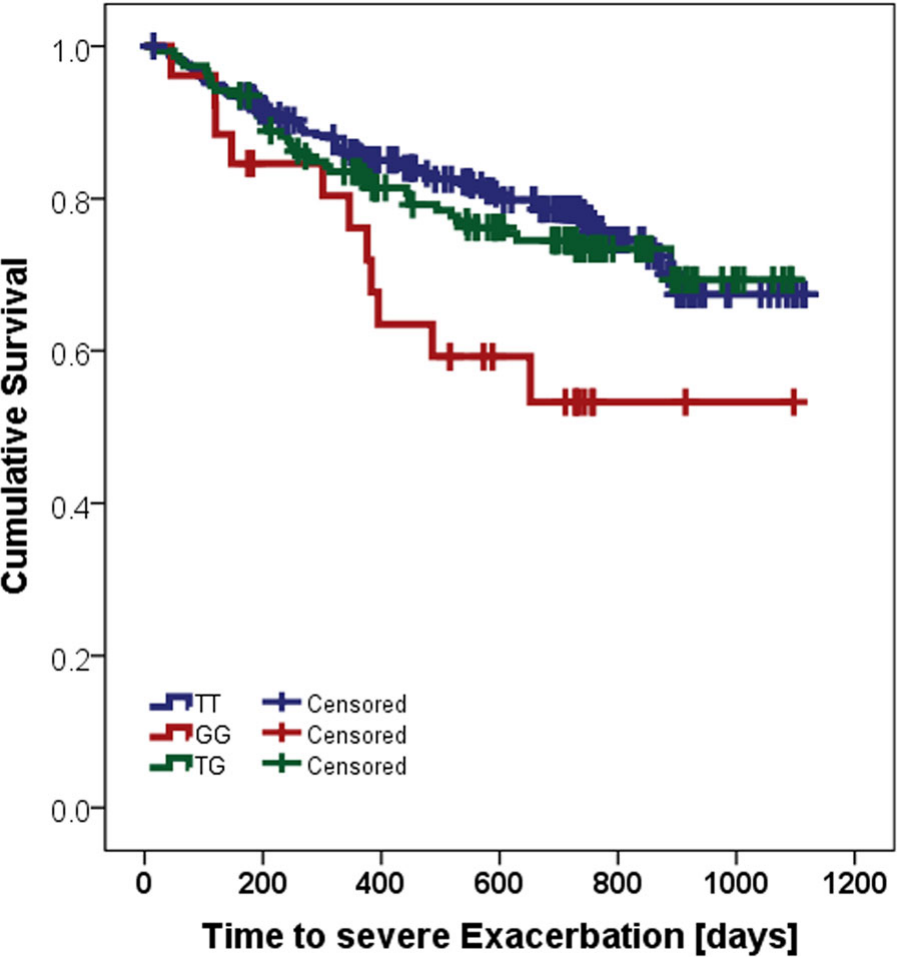
Kaplan Meier curve showing the significant effect of the rs8099917 GG genotype on time to severe exacerbation; *p* = 0.037. GG *p* = 26, 11 events; TT *n =* 335, events = 73; TG *n* = 154, events = 39

Patients with the rs8099917 GG genotype had a significantly shorter time to severe exacerbation than patients with the TT or TG genotype (Fig. 2; *p* = 0.037). Significantly more severe exacerbations occurred in patients with rs8099917 GG genotype compared to patients with rs8099917 TT or rs8099917 TG genotype (42 vs 23%, respectively; chi-squared *p* = 0.032). The rs8099917 genotypes had no significant effect on mortality (*p* = 0.726). There was a significant association between MMRC and the rs8099917 GG genotype with 42% (11/26) of patients with the GG genotype having an MMRC more than the median compared to 22% (107/479) of patients with rs8099917 TT or rs8099917 TG genotype (Table 2; chi-squared, *p* = 0.030). Although there was no difference in the unadjusted Charlson score (*p* = 0.705), suggesting a similar distribution of comorbidities and life expectancy between the groups, five times the number of patients with rs8099917 GG genotype had been diagnosed with a malignancy at the start of the study compared to patients with rs8099917 TT or rs8099917 TG genotype (chi-squared, *p* = 0.014). The association between rs8099917 GG genotype and having a malignancy remained after adjusting for age and smoking (OR = 6.726, *p* = 0.003). Mann-Whitney-U-Test showed a significant difference in C6M between rs8099917 GG genotype and rs8099917 TT/TG genotype (Table 2).

**Table 2:** A comparison of the patient baseline characteristics according to their rs8099917 genotype

	GG (median, IQR)	TT/TG (median, IQR)	*p*-value
Gender: Male (n,%)	16 (61.5)	355 (71.9)	0.269
Age, years	70.50 (14.50)	67.00 (13)	0.139
Current smoker (n, %)	4 (15.4)	149 (30.3)	0.125
PY, months	40.00 (36.75)	45.00 (35)	0.261
BMI, kg/m2	27.29 (7.83)	25.86 (6.14)	0.339
Unadjusted Charlson Score	1.00 (2.00)	1.00 (1.00)	0.305
6MWT, m	375 (161.25)	395 (120)	0.362
Exacerbation rate (number of exacerbations/year)	2.00 (3.00)	1.00 (3.00)	0.935
Severe exacerbation rate (number of severe exacerbations/year)	0.58 (0–3)	0.37 (0–8)	*0.033*
Lung function (post-brd)			
FEV_1_, in L	1.31 (0.8)	1.32 (0.74)	0.749
FVC, in L	2.53 (1.95)	2.69 (1.07)	0.688
FEV_1_/FVC%	49.38 (27.47)	47.00 (22.91)	0.723
FEV_1_, % predicted	56.50 (28.17)	49.50 (25.15)	0.314
FVC, % predicted	82.40 (42.75)	80.00 (26.22)	0.349
BODE index			0.659
≤ median of 3	15 (62.5)	302 (67.3)	
> median of 3	9 (37.5)	147 (32.7)	
Collagen markers [ng/ml]			
C3M	25.3 (14.5)	28.6 (11.6)	0.302
C6M	11.0 (9.7)	13.3 (8.1)	*0.032*
Pro-C3	12.1 (6.1)	10.8 (5.6)	0.684
Pro-C6	8.1 (3.7)	8.0 (0.535)	1.000
GOLD Grade^a^ (n,%)			0.699
GOLD II	15 (57.7)	242 (49.6)	
GOLD III	8 (30.8)	169 (34.6)	
GOLD IV	3 (11.5)	77 (15.8)	
MMRC Test (n,%)			*0.030*
≤ median of 2	15 (57.7)	372 (77.7)	
> median of 2	11 (42.3)	107 (22.3)	
Inflammation markers			
Copeptin, pMol/l	8.24 (10.45)	8.57 (12.53)	0.465
Adrenomedullin, nMol/l	0.60 (0.28)	0.60 (0.3)	0.929
ANP, pMol/l	91.38 (52.43)	83.47 (83.18)	0.408
Procalcitonin, μg/l	0.08 (0.03)	0.08 (0.03)	0.862
SF-36			
Physical function	45 (47.5)	50 (45)	0.346
Role physical	50 (100)	50 (100)	0.742
Role emotional	100 (75)	100 (100)	0.518
Social Functioning	75 (65.6)	75 (50)	0.852
Mental Health	67.50 (36.25)	65 (27.50)	0.780
Body Pain	80 (58)	80 (48)	0.720
Vitality	46.88 (32.81)	50 (31.25)	0.403
General Health	37.50 (42.5)	50 (36.69)	0.570
SGRQ			
Symptoms score	46.60 (26.87)	49.72 (34.44)	0.356
Activity score	66.19 (31.99)	54.43 (31.81)	0.607
Impact score	29.16 (25.68)	29.39 (26.46)	0.802
Total score	44.15 (31.27)	39.03 (27.54)	0.892

Continuous data are shown as median (interquartile range) and categorical variables as No. (%). Italicized *p*-values are statistically significant, ie. *p* < 0.05. *BMI* body mass index, *brd* bronchodilator, *BODE* BMI, airflow obstruction, dyspnea and exercise capacity; *6MWD* 6-min walk distance, *C3M* fragments of collagen type III, *C6M* fragments of collagen type VI, *Pro-C3* pro-forms of collagen type III, *Pro-C6* pro-forms of collagen type VI, *GOLD* Gold Initiative for Chronic Obstructive Lung Disease, *MMRC* modified Medical Research Council, *ANP* Atrial Natriuretic Peptide, *SF-36* 36-item Short-Form Health Survey, *SGRQ* St. George’s Respiratory Questionnaire

^a^GOLD grades are based on FEV1% predicted: 50% ≤ II ≤ 80%; 30% ≤ III ≤ 50%; and IV ≤ 30%

The rs12979860 genotypes had no effect on mortality (*p* = 0.703), exacerbation rate (*p* = 0.946) or time to exacerbation (*p* = 0.324). The rs12979860 CC genotype was associated with BODE index with 46% (30/65) of the patients with a BODE index more than the median compared to 36% (44/122) of patients with the rs12979860 TC and 20.5% (8/39) of patients with the rs12979860 TT genotype (*p* = 0.031).

### Circulating IFNL3 in serum

**Fig. 3:**
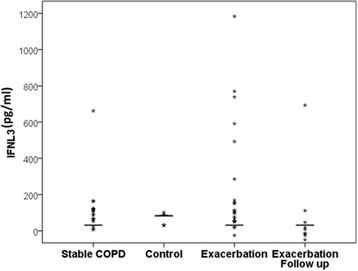
Circulating IFNL3 during stable COPD, in healthy controls, during exacerbation and during follow up to exacerbation 4 weeks later

Circulating IFNL3 was detectable in 3.6% (19/528) of the COPD patients during stable phase and in 7.2% (32/446) of the COPD patients during the exacerbation phase whereas it was detectable in 80% (24/30) of the blood samples from healthy controls. The level of IFNL3 was higher in the controls compared to the COPD patients during stable phase, exacerbation phase and follow-up to the exacerbation (Fig. 3).

There was no association between the rs8099917 (chi-square test; *p* = 0.392) or rs12979860 genotypes (chi-square test; *p* = 0.733) and whether IFNL3 was detectable or not during the stable phase.

### Circulating IFNL3 during stable COPD phase

None of the patients with a GOLD IV classification had detectable circulating IFNL3 during stable phase. 3% (5/180) of the patients classified as GOLD III and 5% (14/262) of the patients classified as GOLD II had detectable circulating IFNL3 levels. GOLD II (31.2 pg/ml; Range = undetectable – 661.83) and GOLD III (31.2 pg/ml; Range = undetectable – 122.26) patients had similar levels of circulating IFNL3, which was less than in healthy controls (82.7 pg/ml; IQR = 81.9–83.4). Using a Mann-Whitney-U-test, patients with detectable circulating IFNL3 had significantly better post-bronchodilator FEV_1_%predicted than patients with undetectable IFNL3 (57.97 vs. 49.62; *p* = 0.035). We found a significant correlation between circulating IFNL3 and post bronchodilator FEV_1_%predicted (Spearman Rho = 0.098; *p* = 0.034). There was no significant difference in other variables, including quality of life, between patients with detectable circulating IFNL3 and patients with non-detectable circulating IFNL3.

### Circulating IFNL3 and collagen biomarkers

There was a significant association between circulating IFNL3 and Pro-C3 (Linear regression, Beta = 0.099 95% CI 0.037–0.634; *p* = 0.028) but no association with C3M, C6Mor Pro-C6. Patients with detectable circulating IFNL3 had significantly more Pro-C3 than patients with undetectable levels of IFNL3 (Mann-Whitney U-Test, median 16.1 [IQR = 8.0] vs 10.7 [IQR = 5.6] ng/ml, respectively; *p* = 0.003). There was no significant difference in the other collagen biomarkers between the two groups of patients.

## Discussion

Viral infections are thought to play a role in the exacerbation of COPD [18, 19]. IFNL3, a member of the interferon lambda family, has immune-modulatory and anti-tumorigenic effects and is induced by viral infections [14]. This is the first study evaluating the association between IFNL circulating levels and its polymorphisms in patients with COPD.

The distribution of both rs8099917 and rs12979860 SNPs adhered to the Hardy Weinberg Equilibrium. Ethnic background strongly impacts SNP distribution, however, people with Caucasian ancestry (the main ethnic background of the present cohort) have a more balanced distribution of genetic polymorphisms [9, 34]. In our COPD cohort, the SNP rs8099917 GG genotype significantly influenced the severe exacerbation rate, and the time to severe exacerbation and it was associated with a higher MMRC score. Conversely, SNP rs12979860 had no effect on exacerbations or death. This is in line with what is known for hepatitis and diabetes, where the SNP rs8099917 GG genotype is considered to be the risk genotype and the SNP rs12979860 CC genotype the protective genotype [35–37]. The IFNL3 polymorphisms predict response to treatment in patients with hepatitis C [27, 30, 31]. We found that the prevalence of malignancy was increased among patients with the rs8099917 GG genotype. This was also seen in patients with chronic hepatitis C, where more patients with the rs8099917 GG or non-TT genotype had hepatocellular carcinoma [38]. In antiretroviral-treated HIV-infected patients, however, the SNP rs12979860 CC genotype was associated with higher mortality and thus it was not protective [39]. In COPD patients, we found that the SNP rs12979860 CC genotype was associated with a higher BODE index, and thus possibly a higher risk of mortality, though the rs12979860 CC genotype did not associate with mortality directly in this study.

We found no association between the genotype and the circulating IFNL3 levels. In the literature, the association between genotype and circulating IFNL3 levels varies according to illness and group. Arpaci et al. [40] found no association between genotype and circulating IFNL3 in patients with Hashimoto’s Thyroiditis. Langhans et al. [41] found that hepatitis C patients with the protective SNP rs12979860 CC genotype had more circulating IFNL3 compared to patients with the SNP rs12979860 TT genotype whereas Alborzi A., et al. [42] found no association between circulating IFNL3 and genotype.

Less IFNL3 is secreted by primary cells from asthmatic patients compared to cells from healthy controls infected with a virus but the basal levels are similar between the two groups [22]. Bullens et al. [43] found increased basal IFNL3 mRNA in the sputum of asthmatic patients compared to controls. Thus far there is no literature regarding basal serum IFNL3 levels in asthma patients compared to controls. Circulating IFNL3 levels were similar between hepatitis C patients and healthy controls [41] and Arpaci et al. [40] found increased basal circulating IFNL3 in patients with Hashimoto’s thyroiditis compared to healthy controls. In our cohort of COPD patients, basal circulating IFNL3 levels were significantly less compared to controls. The circulating IFNL3 levels increased during an exacerbation, as was also seen in vitro in the cells from asthmatic patients that were infected with virus [22], and then returned to basal levels after the patient had recovered from the exacerbation. In COPD, the basal circulating IFNL3 levels were associated with the severity of airflow limitation. We hypothesise that the difference in circulating IFNL3 between healthy donors, GOLD II, GOLD III and GOLD IV patients may be due not only to the association between IFNL3 and FEV1, but also due to remodelling of the extracellular matrix in the lungs. This hypothesis is in part corroborated by the fact that there is a strong association between circulating IFNL3 and Pro-C3. Pro-C3 is the N-protease cleavage site of type III collagen and is a marker of tissue formation [44]. Low levels of Pro-C3 is associated with worse lung function [31, 45] and with a shorter time to severe exacerbation [31]. It is therefore possible that changes in the cell structure of the lung results in decreased secreted IFNL3 which causes an impaired immune response to infection. More exacerbations occur resulting in more remodelling of the cells and less IFNL3 secretion, both of which are associated with impaired lung function, and a vicious cycle is continued. It is also possible that the decreased circulating IFNL3 facilitates viral infection leading to less Pro-C3 which results in worse lung function and shorter time to exacerbation. We are unable to determine which element is the catalyst therefore further studies are required to explore the association between IFNL3 polymorphisms and the remodelling of the extra-cellular matrix in stable and exacerbated COPD.

We found no association between circulating IFNL3 levels and disease outcome as is also evident in patients with hepatitis C [41].

The main limitation to this study is that there are no genotyping results for healthy controls and the SNP data was not validated in a separate cohort. We only investigated two SNPs, further studies are needed to investigate other SNPs related to IFNL3. In addition, the circulating IFNL3 was measured from unmatched blood donor samples. However, we found no association between circulating IFNL3 levels and gender or age, so the differences seen between the healthy controls and the COPD patients probably are not due to gender or age differences. The clinical value of IFNL3 alone or in combination with other biomarkers has to be assessed in conformational and randomized clinical trials.

Strengths of the study include the originality, longitudinal design assessing clinically relevant end-points and the fact that both genotypes and circulating IFNL3 were determined in a large multicentric cohort.

## Conclusions

IFNL3 polymorphisms may play a role in disease activity and outcomes in COPD and circulating IFNL3 may be associated with disease severity and stability. Further investigations are required to determine the underlying mechanisms.

## Abbreviations

6MWT: 6 Minute walk test
ADM: Pro-adrenomedullin
ANP: Atrial natriuretic polypeptide
BMI: Body mass index
BODE: Body mass index, airflow obstruction, dyspnea and exercise capacity
C3M: Collagen type III
C6M: Collagen type VI
GOLD: Gold Initiative for Chronic Obstructive Lung Disease
IFNL: Interferon lambda
MMRC: Modified medical research council
Pro-C3: Pro-form of collagen type III
Pro-C6: Pro-form of collagen type VI
QoL: Quality of life
SNPs: Single nucleotide polymorphisms
